# Optimization of R-Phycoerythrin Extraction by Ultrasound-Assisted Enzymatic Hydrolysis: A Comprehensive Study on the Wet Seaweed *Grateloupia turuturu*

**DOI:** 10.3390/md21040213

**Published:** 2023-03-28

**Authors:** Cécile Le Guillard, Jean-Pascal Bergé, Claire Donnay-Moreno, Josiane Cornet, Jean-Yves Ragon, Joël Fleurence, Justine Dumay

**Affiliations:** 1 IFREMER Centre Ifremer Atlantique, EM3B, BP 21105, CEDEX 03, 44311 Nantes, France; 2 Institut des Substances et Organismes de la Mer, ISOMER, Nantes Université, UR 2160, 44000 Nantes, France; 3 UPCYCLINK, 22 Rue de Mangorvenec, 56890 Saint-Avé, France

**Keywords:** seaweed, *Grateloupia turuturu*, R-phycoerythrin, ultrasound-assisted enzymatic hydrolysis (UAEH), extraction process, response surface methodology

## Abstract

Enzyme-assisted extraction (EAE) and ultrasound-assisted extraction (UAE) are both recognized as sustainable processes, but little has been done on the combined process known as ultrasound-assisted enzymatic hydrolysis (UAEH), and even less on seaweed. The present study aimed to optimize the UAEH of the red seaweed *Grateloupia turuturu* for the extraction of R-phycoerythrin (R-PE) directly from the wet biomass by applying a response surface methodology based on a central composite design. Three parameters were studied: the power of ultrasound, the temperature and the flow rate in the experimental system. Data analysis demonstrated that only the temperature had a significant and negative effect on the R-PE extraction yield. Under the optimized conditions, the R-PE kinetic yield reached a plateau between 90 and 210 min, with a yield of 4.28 ± 0.09 mg·g^−1^ dry weight (dw) at 180 min, corresponding to a yield 2.3 times higher than with the conventional phosphate buffer extraction on freeze-dried *G. turuturu*. Furthermore, the increased release of R-PE, carbohydrates, carbon and nitrogen can be associated with the degradation of *G. turuturu* constitutive polysaccharides, as their average molecular weights had been divided by 2.2 in 210 min. Our results thus demonstrated that an optimized UAEH is an efficient method to extract R-PE from wet *G. turuturu* without the need for expensive pre-treatment steps found in the conventional extraction. UAEH represents a promising and sustainable approach that should be investigated on biomasses where the recovery of added-value compounds needs to be improved.

## 1. Introduction

Since the 1970s, seaweed production has been largely dominated by aquaculture, representing 97% of the global production in 2019 (34.7 million tonnes of wet weight). Red seaweeds are the main cultivated phylum, representing approximately 52.6% of the global aquaculture production of seaweed in 2019 (18.3 million tonnes of wet weight) [1]. Some of them have been used for hundreds of years for various applications, such as for human consumption (sea vegetable), animal feed and fertilizer, and since the 1940s they have been used in industry for the production of hydrocolloids [2,3]. Currently, most of the red seaweeds remain underexploited, especially in Europe where the consumption of seaweed as food is regulated. Only a few species are therefore approved as sea vegetables or ingredients; they are listed in the European Novel Food Catalogue (Novel Food Regulation (EU) 2015/2283) [4].

*Grateloupia turuturu* is a polymorphic red seaweed belonging to the order of Halymeniales. It is a native species from Japan, and while commonly consumed in Asia, it is not yet part of the authorized species in Europe. This non-indigenous species was introduced in Brittany, the first record of which dates from 1989, and is considered at the moment as potentially invasive on the French Atlantic coasts (Brittany) [5] as well as in Portugal where its distribution has some typical features of an invasive organism [6]. However, this available biomass remains underexploited in spite of its content in compounds of interest. Some studies have already demonstrated that *G. turuturu* could have a great potential of exploitation in aquaculture, nutraceutical, pharmaceutical and cosmeceutical industries [5,7,8,9,10]. Indeed, it could be a source of nutritional compounds for humans and animals, with up to approximately 60% of total dietary fibres [11], 27% dw of total proteins [12], including all the essential amino acids for 38% of total amino acids [13], and around 5% of total lipids [14], among which the presence of polyunsaturated fatty acids (PUFA) which are mainly represented by eicosapentaenoic acid (ω3 PUFA) [15]. *G. turuturu* is also a source of potential bioactive molecules, such as anti-microfouling and anti-bacterial, as well as high added-value compounds such as the R-phycoerythrin pigment (R-PE) [5,6,10,16]. Additionally, previous studies reported that its biochemical composition is affected by environmental factors, notably seasonal and geographical parameters [10,11,12,14].

R-phycoerythrin belongs to a family of light-harvesting pigment proteins, named phycobiliproteins. It is responsible for the red-pink colour of red seaweeds by masking the green pigment chlorophyll [17,18]. This accessory pigment is the most abundant phycobiliprotein in Rhodophyta and consists of one proteinic part covalently linked to a chromophore to form three subunit types α, β and γ. Theses subunits are associated to give the hexameric structure of R-PE, with a molecular weight ranging from 240 to 260 kDa. The R-PE possesses specific spectral properties: it notably absorbs the light in the yellow-green spectral range (between 498 and 565 nm) with absorbance maxima at 565 nm, 540 nm and 498 nm, and it has a fluorescence emission maximum at around 575 nm [17,19,20]. This pigment is already used as a fluorescent probe in advanced biotechnologies, as a natural food colorant notably in Asian countries, and it exhibits several bioactivities which could be exploited for the pharmaceutical field [3,19,21,22]. Due to these various potential markets, some previous studies have more particularly investigated the extraction and the pre-purification of R-PE from *G. turuturu* as a way to exploit this available biomass [23,24,25,26]. Thus, it has been demonstrated that using a conventional extraction with sodium phosphate buffer on freeze-dried *G. turuturu*, the R-PE extraction yield can reach up to 5.28 mg·g^−1^ dw [23].

The conventional extraction method used on *G. turuturu* is performed on freeze-dried thalli ground in liquid nitrogen and suspended in a phosphate buffer solution [23]. On some conventional extracts it has been demonstrated that the use of precipitation, ultrafiltration and anion exchange chromatography techniques was efficient for the partial purification or purification of the R-PE, notably from *G. turuturu* [23,25,26,27].

However, alternative and innovative methods would be valuable, notably to avoid expensive pre-treatments, and to save time and increase the extraction of other compounds. In this context, enzyme-assisted extraction (EAE) has demonstrated its interest for R-PE extraction from various red seaweed species [28,29,30], but without success on *G. turuturu* [31]. Over the past two decades, different innovative techniques such as microwave-assisted extraction, supercritical fluid extraction and pressurized liquid extraction, have emerged to retrieve biomolecules from seaweeds [32,33,34]. Our previous studies applied, for the first time, to the wet *G. turuturu* one of those alternative techniques, namely ultrasound-assisted extraction (UAE) and ultrasound-assisted enzymatic hydrolysis (UAEH), to extract water soluble compounds such as R-PE [13,35]. Based on the literature reviews of the last ten years, it appears that UAE is a promising and efficient process to extract plant pigments [36,37,38] and seaweed compounds [39,40,41,42,43,44,45], including phycobiliproteins [45,46]. Regarding the UAEH, despite an increase in the use of this combined process on various biomasses of plant origin (i.e., pumpkin, cassava waste, goji fruit and sugarcane bagasse) [47,48,49,50], only a few studies have applied it to seaweeds, and they demonstrated promising results [13,35,51,52]. However, UAEH requires further study because it is still difficult to understand the positive effect and underlying mechanisms of ultrasound on enzymatic hydrolysis. It appears to be generally accepted that during UAEH the positive effects of ultrasound can be attributed to the turbulence and the mass transfer caused by the implosion of cavitation bubbles, thereby increasing the access of the substrate to the enzyme [50,53,54,55]. In addition, the efficiency of this combined process would be influenced by numerous operating parameters related to enzymes, free or immobilized, and ultrasound devices [38,53,55,56] that need to be systemically investigated and optimized. Previous works have already demonstrated that experimental designs are an efficient tool to improve the R-PE extraction yields from other red seaweeds by enzymatic hydrolysis [28,29,30], to optimize the UAE of compounds from *Ascophyllum nodosum* [44,57], and the UAEH of compounds of plant and animal origin such as bioactive polysaccharides from a plant leaves and molluscs [58,59]. 

The aim of the present work was to evaluate the potential of UAEH to extract R-PE from wet *G. turuturu.* To this end, we used a central composite design to study the effects of three UAEH parameters on the R-PE extraction yields: power, temperature and flow rate. We demonstrated that after optimization of these parameters the extraction yield of R-PE was significantly increased in comparison with the conventional extraction on freeze-dried *G. turuturu*. This research article highlights the potential of this combined process to extract high added-value compounds from biomasses recalcitrant to enzymatic hydrolysis, as well as carbohydrates, carbon and nitrogen. 

## 2. Results and Discussion

### 2.1. Experimental Design

The R-PE extraction yields obtained for each experiment are reported in Table 1. The experiment N° 2, corresponding to the lowest level of temperature (20 °C) and medium levels of power (300 W) and flow rate (145 L·h^−1^) led to the highest R-PE extraction yield. Under these conditions, a marked increase of the R-PE in the first hour can be observed, reaching up to 4.19 mg·g^−1^ at 180 min, followed by a very slow decrease (4.06 mg·g^−1^ at 360 min). Conversely, for experiment N° 7, in the same conditions of power and flow rate (300 W and 145 L·h^−1^) but with the highest temperature (40 °C), the lowest R-PE extraction yields were obtained over time, with 2.42 mg·g^−1^ at 180 min, which represents a difference of 1.7 times between experiment N° 2 and N° 7. This result is consistent with our previous study demonstrating that a high temperature (40 °C) negatively affects the extraction of R-PE by the UAEH process [35], and with previous studies demonstrating the thermal sensitivity of this pigment [24,35,60]. Furthermore, all the experiments carried out at 36 °C (experiment N°s 10, 12, 14, 18) led to low R-PE extraction yields, relatively stable over time: between 2.39 (experiment N° 14 at 180 min) and 2.92 mg·g^−1^ (Experiment N° 18 at 60 min). According to these results, it seems that it will be suitable to carry out the UAEH of *G. turuturu* at the lowest level of temperature (20 °C), and the levels of power and flow rate seem not to have a strong effect on the R-PE contents. These trends will then be checked by the data analysis of the experiment design.

### 2.2. Predicted R-PE Extraction Yields

**Figure 1:**
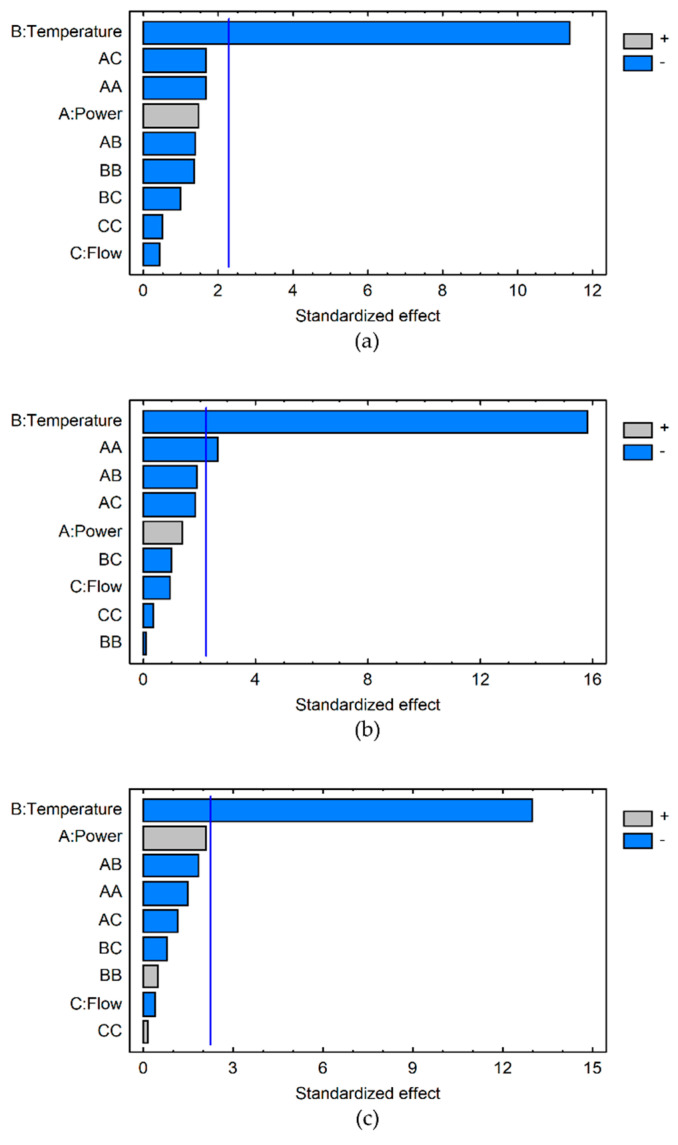
Pareto charts obtained at 120 min (**a**), 180 min (**b**) and 240 min (**c**) for the analysis of R-PE extraction using the UAEH process according to temperature (B), ultrasonic power (A) and flow rate (C) conditions. The significance level, with *p* value = 0.05, is represented by the vertical line. The light grey and blue bars represent positive and negative effects, respectively.

The R-PE extraction yields obtained for the 19 experiments were analysed independently for each point of the kinetics (0, 30, 60, 120, 180, 240, 300, 360 min) (Table 1). The data showed that the highest R-PE extraction yields were obtained for experiments between 120 and 240 min, for which a canonical analysis has been performed. The influence of each variable on the R-PE extraction yield was determined. As illustrated by Pareto charts (Figure 1a–c), at 120, 180 and 240 min the temperature increase has a significant negative effect on the R-PE extraction yield (*p* < 0.001). At 120 min and 240 min, the temperature is the only variable significantly affecting the extraction of R-PE, especially temperature increase, which results in a negative effect. This confirms the trend noticed in our previous study conducted at 22 °C and 40 °C [35]. At 180 min (Figure 1b), in addition to this significant negative effect of the temperature (B) (*p* < 0.001), a negative quadratic effect of the ultrasound power (AA) is observed (*p* < 0.05). Despite this quadratic effect, it appears that the ultrasonic power does not have a significant effect on the R-PE extraction yield in the tested conditions. The influence of the ultrasonic power is complex. Some studies conducted on different biomass and in other experimental conditions of UAE or UAEH have previously demonstrated that a power increase improved the extraction of pigments up to a critical power level, from which the inverse effect was observed with a decrease of pigment extraction yields [36,37,61]. The same observations have been depicted for the UAEH of polysaccharides from plant materials [47,49,58] and molluscs [59]. Three factors are believed to explain this phenomenon: too many cavitation bubbles, which would reduce the energy transmission into the medium [36]; coalescence of cavitation bubbles, which would implode less strongly [37]; and the inhibition of the enzymatic activities [61]. Regarding the flow rate (Figure 1), it never has a significant effect on our UAEH process. With the exception of a few publications, such as Zhao et al. working on microalgae sonication [62], this parameter has been very under studied in the literature because processes involving ultrasonic technologies used mainly batch reactors without a recirculating loop. It has to be kept in mind that comparison between studies is quite difficult due to the ultrasound parameters which present numerous differences, such as the kind of ultrasonic reactor (more often ultrasonic baths or probes), the ultrasonic frequency and intensity applied, the temperature of the medium, the solvent and matrix properties, and how the process is carried out (open or close/batch reactor) [38,63]. This is even more difficult for the UAEH process due to additional parameters induced by the presence of different enzymes (type of enzymes, pH, temperature, enzyme/substrate ratio, etc) and the interactions between ultrasound and enzymes [53].

After removing the insignificant effects, the equations of the model at 120, 180 and 240 min were obtained (Table 2). The adjusted R² values comprised between 85 and 92% indicate a high robustness of the extraction pattern of the R-PE using the UAEH process. The UAEH process for the extraction of R-PE was thus optimized for these three kinetic points. Remarkably, our model predicted identical optimized conditions at 120, 180 and 240 min (Table 2): 300 W, 20 °C, 145 L·h^−1^. These conditions are consistent with those that allowed us to reach the highest R-PE extraction yield during the experimental design (Experiment N° 2, 4.19 mg·g^−1^ dw at 180 min) (Table 1). Although the optimal operating temperature is lower than the recommendations of the enzyme’s suppliers, previous studies have shown that 24 °C is the optimized temperature for the enzymatic extraction of R-PE from *Palmaria palmata* against the 40 °C recommended by the supplier for the working activity of its enzyme [28]. It also demonstrates that a moderate temperature is preferable as the R-PE is well known to be a heat-sensitive pigment [24,35,60].

The highest extraction yield was predicted at 180 min with 4.32 mg·g^−1^ dw; a slightly lower value was predicted at 120 and 240 min with 4.21 mg·g^−1^ dw. Once again, a similar trend was observed in experiment N° 2 (Table 1). Thus, it can be assumed that the highest R-PE content was reached at approximately 180 min. It was therefore decided to increase the number of kinetics points, every 30 min after 90 min, in order to refine the kinetic. The UAEH were carried out over 210 min to avoid a decrease of the R-PE content in the soluble fraction. Indeed, the duration of exposure to ultrasonic waves is an important parameter to consider, particularly when sensitive compounds are targeted [38], such as natural pigments [36,64]. Hence, it appears relevant to refine the kinetic to improve its accuracy. The relevance of the optimized conditions was further tested by conducting UAEH experiments in three independent replicates (n = 3), at 300 W, 20 °C and 145 L·h^−1^.

### 2.3. Validation of the Predicted R-PE Extraction Yield: UAEH Conducted under Optimized Conditions

#### 2.3.1. R-Phycoerythrin Content

**Figure 2:**
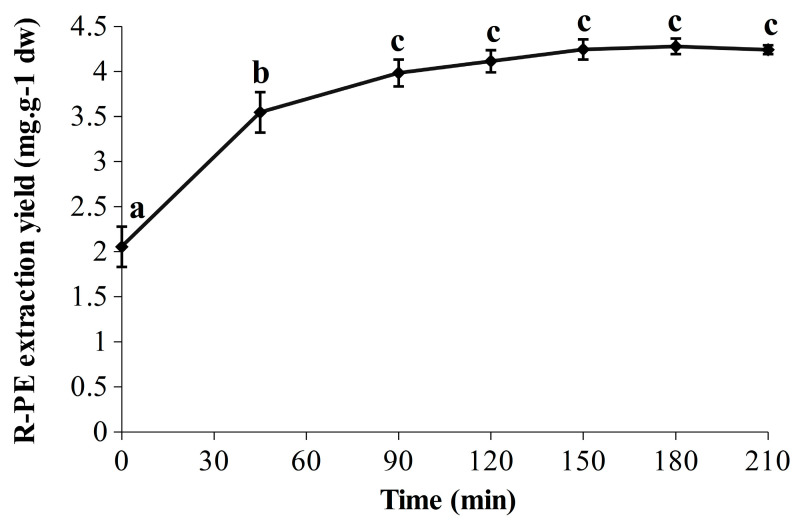
Evolution of the R-PE extraction yield (210 min) in optimized conditions of ultrasound-assisted enzymatic hydrolysis (UAEH) (300 W, 20 °C, 145 L·h^−1^). Values are means ± SD from three independent experiments (n = 3). Significant differences (*p* < 0.05) are indicated by different letters.

The R-PE extraction yields obtained over time are represented in Figure 2. A significant marked increase was observed in the first 45 min (from 2.05 ± 0.22 to 3.55 ± 0.22 mg·g^−1^ dw), followed by a slower but significant increase up to 90 min (3.98 ± 0.15 mg·g^−1^ dw), leading to a plateau. The statistically predicted extraction yields at 120, 180 and 240 min were 4.21, 4.32 and 4.21 mg·g^−1^ dw, respectively, which is perfectly in line with the experimental results. Indeed, the highest extraction yield was observed at 180 min with 4.28 ± 0.09 mg·g^−1^ dw, whereas the values were slightly lower at 120 min and 210 min with 4.11 ± 0.12 mg·g^−1^ dw and 4.24 ± 0.05 mg·g^−1^ dw, respectively. In addition, the achievement of a refined kinetic was relevant as it allows one to see the break in the extraction yield between 45 and 90 min, and also that R-PE extraction yields were similar (no significant differences) from 90 to 210 min (included). The R-PE yield reached at 90 min was stable for at least 2 h (corresponding to the end of the extraction) and that could be true for even longer according to the results of experiment N° 2 (Table 1). Thus, in these conditions, the soluble R-PE seems to be relatively stable under ultrasonic waves over time. Comparison with classical maceration extraction has been performed to ensure the efficiency of this UAEH process. Thus, the optimized UAEH allowed us to reach a higher R-PE extraction yield (4.28 ± 0.09 mg·g^−1^ dw after 180 min (Figure 2)) than the conventional method in sodium phosphate buffer (20 mM; pH 7.1) (1.90 ± 0.03 mg·g^−1^ dw) while avoiding freeze-drying and grinding in liquid nitrogen, which enables savings in time and money. This part of our study demonstrates once again that UAEH is an efficient and promising alternative to the conventional technique to extract R-PE from *G. turuturu*. Finally, for future works, thorough analyses would have to be carried out during UAEH to verify the integrity and properties of R-PE through fluorescence measurements, electrophoresis and chromatographic methods; after that, a pre-purification could be considered [12,23,27,60].

#### 2.3.2. Seaweed Liquefaction

**Figure 3:**
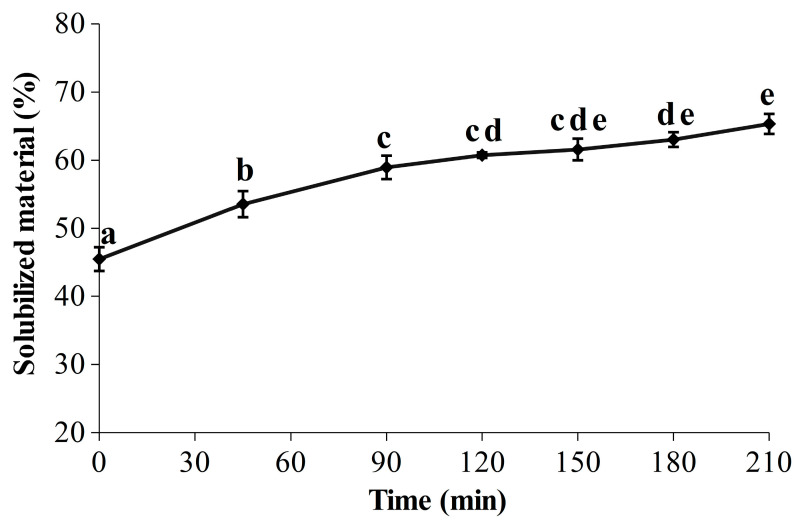
Evolution of the seaweed liquefaction in optimized conditions for the extraction of R-PE by ultrasound-assisted enzymatic hydrolysis (UAEH) (300 W, 20 °C, 145 L·h^−1^); results are the percentages of solubilized material over time (210 min). Values are means ± SD from three independent experiments (n = 3). Significant differences (*p* < 0.05) are indicated by different letters.

Red seaweeds are a promising biomass for an integrated biorefinery approach due to their worldwide distribution and their content in a wide range of chemical components, such as minerals, vitamins, lipids, carbohydrates, proteins and high value-added pigments (R-phycoerythrin and R-phycocyanin) [65]. Thus, R-PE is most likely not the only compound of interest contained in the soluble fractions, and the seaweed liquefaction over time has therefore been evaluated (Figure 3). Looking at the results, it is apparent that the start of the kinetic shape for the liquefaction is close to the one obtained for the R-PE yield (Figure 2), with a significant and marked increase during the first 90 min, from 45.50 ± 1.74% to 58.96 ± 1.72%. A smaller but significant increase between 90 min (58.96 ± 1.72%) and 180 min (63.04 ± 1.08%) was then measured, indicating that the seaweed liquefaction slowed down. At 210 min, 65.34 ± 1.46% of the seaweed compounds were released in the soluble phase, corresponding to an increase of 1.4 times in comparison with the initial level. Kadam et al. demonstrated that ultrasound improved the liquefaction of *Ascophyllum nodosum* with a strong correlation between the protein extraction yield and the percentage of solubilized material, as seen here for our protein pigment [41]. Since the R-PE yield appeared quite stable over time (Figure 2), it would therefore be interesting and advisable to pursue the UAEH up to 90 min for the recovery of other valuable molecules in a biorefinery approach. Further biochemical analyses at the end of UAEH (210 min) were thus carried out, and the results are described in the next section (2.3.3). However, because the liquefaction had also been investigated in a previous study [35], we first compared the results obtained here and in our previous work. As expected, after 210 min in the optimized conditions for the R-PE extraction, the percentage of solubilized material appeared sensitively lower than the one obtained after only 180 min in harsher conditions of power and temperature (400 W and 40 °C) (86.37 ± 1.43%); this is also the case for a temperature closer to the one used in the present study (400 W and 22 °C) (76.75 ± 2.44%). By showing that a reduction of power from 400 W to 300 W clearly decreased the rate and level of liquefaction, this study provides novel clues regarding the effect of the ultrasonic power on seaweed liquefaction by UAEH. This is in line with some studies investigating the UAE process to recover bioactive compounds from brown and red seaweed species, where the highest extraction yields were achieved by operating at the highest ultrasonic powers tested [39,44,66]. Our results here with an UAEH process applied on wet *G. turuturu* show a similar trend, where higher ultrasonic power increases the percentage of solubilized material.

#### 2.3.3. Further Biochemical Analyses of Optimized Soluble Fractions

Biochemical analyses of soluble fractions have been carried out at the beginning (T0 min) and at the end of the extraction period (T210 min) in order to characterize the solubilisation of the different compounds of *G. turuturu* as well as their recovery levels. The results reported in Table 3 have been corrected by removing from soluble fractions the amount of carbon, nitrogen and carbohydrates provided by the enzymatic cocktail. Thus, in these conditions and at the end of optimized UAEH, nitrogen and carbon extraction yields reached 55.51 ± 1.36% and 48.76 ± 1.53%, respectively. As with the percentage of solubilized material, which was multiplied by 1.4 after 210 min, nitrogen and carbon extraction yields also increased significantly over time, because they were multiplied by factors of 1.7 and 2.1, respectively. Regarding nitrogen, the use and the efficiency of enzymes (proteases and/or carbohydrases) for the extraction of nitrogen compounds from seaweeds has been well documented [28,67,68,69,70,71], whereas the use of ultrasound with or without enzymes is more recent [13,39,40,41,52]. However, our results indicate that the UAEH process applied on wet *G. turuturu* is also very efficient for the carbon release, which has not been well documented yet. Indeed, the carbohydrate content in the soluble phase has significantly increased by 3.2-fold (141.86 ± 20.86 mg·g^−1^ dw after 210 min). This result is not surprising, knowing that the enzymes used here are dedicated to the hydrolysis of polysaccharides (glycosidases) and ultrasound have already been shown to intensify the enzymatic hydrolysis of various biomass for the release of carbohydrates and polysaccharides [40,47,48,50,51,54,59]. It is therefore logical that, when combined, these two processes lead to a higher release of carbon in the soluble phase. Despite the limitations preventing the direct comparison of two studies, it is important to remember that, in our previous research work on *G. turuturu* [13], after 360 min of UAEH (400 W, 22 °C, 50 L·h^−1^), the content of soluble carbohydrates was higher, reaching 210 ± 14 mg·g^-1^ dw. Based on these two studies, it appears clear that UAEH parameters need to be optimized according to the nature of the targeted compounds, and they have to be carefully considered beforehand.

The carbohydrate content in the extracts was further characterized by determining the weight-average molecular weight (Mw) of the polysaccharides at T0 and T210 min (Table 3). Of note, the average Mw value of the carbohydrates contained in the enzymatic preparations has also been investigated, and the Mw appears to be much lower and clearly distinctive of the Mw obtained at T0 and T210 for extracted polysaccharides. At the beginning of the extraction, a high Mw was measured with a value of 1080 ± 51 kDa. Although the polysaccharides composing *G. turuturu* are not well known, some studies reported the presence of cellulose, a small fraction of agar, and more recently hybrid kappa/iota/theta carrageenans in the cell wall of this species [6,31]. This value can thus be compared to the average Mw values of carrageenans ranging from 260 kDa for kappa carrageenans to 1400 kDa for iota carrageenans [72], and the results obtained here seem to be in accordance with the presence of hybrid kappa/iota carrageenans.

Regarding the Mw evolution over time (Table 3), a significant decrease of Mw occurred during our UAEH process from 1080 ± 51 kDa at T0 to 493 ± 24 kDa at T210 min, a 2.2-fold reduction of the Mw. To explain this result, we hypothesize that high Mw polysaccharides were released in the soluble phase at the beginning of UAEH, and those were subsequently degraded by glycosidases as well as ultrasound. Indeed, it has been demonstrated that 15 min of UAE enabled the extraction of high molecular weight laminarins [42], and other studies have demonstrated that sonication enabled polysaccharide depolymerization [59,73,74,75], including brown and red seaweed polysaccharides [43,76,77]. Contrary to the enzymatic hydrolysis of cell wall polysaccharides, in which many parameters have to be optimized (i.e., type of enzyme, enzyme/substrate ratio) for each seaweed specie [69], sonication does not need a thorough knowledge of the polysaccharide structure to induce its degradation [73]. Furthermore, this degradation of *G. turuturu* polysaccharides over time could explain in part the increase of carbohydrates, carbon and nitrogen compounds in the soluble fractions (Table 3), as well as the R-PE, because cell wall polysaccharides are one of the main barriers to the solubilisation of protein components from seaweeds [69].

Regarding the pigment content, we assumed in a previous study [35] that co-extracted compounds, notably carbohydrates, could be involved in the stability of the R-PE yield, and the results obtained here are interesting in that regard.. Indeed, a previous study conducted in our laboratory demonstrated that among the different food preservatives tested, sucrose and ascorbic acid were the most efficient to preserve R-PE [12]. Ascorbic is well known for its antioxidative properties, a patent has demonstrated that it allowed photo-stabilization of phycoerythrin retrieved from *Porphyridium cruentum* [78]. Thus, it can be assumed that natural bioactive compounds from seaweed, such as antioxidative polysaccharides [79], could contribute to R-PE preservation. Moreover, the antioxidant activity of seaweed polysaccharides can be improved by enzymatic hydrolysis [80] and sonication [76,81], through their Mw decrease [76,80,81], and this is even more true with the UAEH process [59]. The assumed link between R-PE yield and co-extracted bioactive carbohydrates could be evaluated in the future by assessing the antioxidant properties of our soluble fractions.

## 3. Materials and methods

### 3.1. Materials

Seaweeds, *G. turuturu*, were harvested on 30 April 2014, in the intertidal zone of Batz-sur-Mer on the Atlantic coast, France. Epiphytes were removed by hand and algae were partially dewatered with a spin-dryer, then vacuum-packed (Boulanger INV 40) and immediately frozen in plastic bags stored at −20 °C in darkness. Algae were used for experiments within the year after the date of harvest. A part of *G. turuturu* was also freeze-dried after harvesting to perform conventional extraction of R-PE. For UAEH, four industrial carbohydrase preparations were used and combined according to their similar pH and temperature optima and their complementarity [13,35]. The enzymatic cocktail was thus composed of Sumizyme TG and Sumizyme MC, produced by SHIN NIHON CHEMICAL and kindly provided by Takabio (Beaucouzé, France); Multifect^®^ CX 15 L, kindly provided by DuPont™ (Wilmington, DE, USA); and Ultraflo^®^ XL, kindly provided by Novozymes^®^ (Bagsværd, Denmark). The ultrasonic flow-through reactor (SONITUBE^®^ 35 kHz, 200 to 400 W) was manufactured and kindly provided by SYNETUDE (Chambéry, France).

### 3.2. Ultrasound-Assisted Enzymatic Hydrolysis (UAEH)

#### 3.2.1. Experimental System

The extraction of R-PE requires one to take into consideration some parameters affecting its stability. The R-PE is stable in a wide range of pH [24,60,82] from pH 3 to 10 for the R-PE extracted from *G. turuturu* [24]. However, it is very sensitive to light [24] and heat, thus it is recommended to not expose it to a temperature higher than 40–60 °C or less (30 °C) depending on the duration of exposure and pH [24,35,60,83].

Prior to UAEH experiments, seaweeds were cut into small pieces (approximately 5–7 mm^2^) using a cutting mill (Microcut Stephan MC 15, Germany) and subsequently stored at −20 °C. According to the process developed in a previous study [35], all the experiments were performed in a jacketed glass reactor vessel (5 L) containing approximately 3 kg of reaction mixture, composed of 20% wet and cut seaweed homogenized in tap water (corresponding to the minimal water quantity to obtain an effective circulation of the reaction mixture) with the pH adjusted to 5.5 by the addition of 6 M HCl (Radiometer analytical TitraLab^®^ 854, HACH^®^, Loveland, CO, USA). Homogenization was conducted continuously at 100 rpm (Stuart^®^ Overhead Stirrer SS20, Bibby Scientific Ltd., Stone, UK), and the reaction mixture was circulated using a peristaltic pump (Heidolph PD 5006—SP standard). An external circulation system (Hitema^®^ ESE 010, Bovolenta Padua, Italy and Memmert, Schwabach, Germany) was used to control and adjust the temperature in the reactor during the 6 h of the process. To ensure R-PE preservation, the whole system was kept in darkness.

The UAEH was initiated by the application of ultrasound (SONITUBE®—SYNETUDE, Chambéry, France—turned on) and the simultaneous addition of the enzymatic cocktail in the reaction mixture. One percent *w/w* of each enzymatic preparation related to the weight of wet seaweed was added. Experiments were monitored for up to 360 min, and regular sampling (± 30 mL) was carried out throughout the experiment. Samples were immediately centrifuged (15,500× *g*, 30 min, 20 °C, Beckman Coulter Avanti^®^ J-E Centrifuge) providing supernatant and sludge fractions that were weighed and then freeze-dried. The temperature was regulated, and pH was monitored inside the reactor continuously during the whole experiment.

#### 3.2.2. Experimental Design

Response surface methodology (RSM) was used to investigate the influence of three variables of ultrasound-assisted enzymatic hydrolysis on the R-PE extraction. The temperature (T) was chosen because R-PE is known to be heat-sensitive and it is also a variable affecting enzyme activities, the power of ultrasound (P) was studied as its effect has never been, to the best of our knowledge, studied for the R-PE extraction, and finally the flow rate in the experimental system (Q), because it impacts the length of stay of seaweed and enzymes in the SONITUBE, thus impacting how long they are subjected to ultrasonic waves. For a second time, these three variables were optimized. A 2^3^ central composite design (CCD) was conducted, each variable taking 5 levels: −α, −1, 0, +1, +α. Star points (−α and +α) were varied systematically at high (*Max*) and low (*Min*) levels. The real values of the factorial points (−1 and +1) were calculated for each variable according to Equation (1) and (2):


(1) $$-1=\frac{\mathrm{Min}+\mathrm{Max}}{2}-\frac{1}{\alpha }(\mathrm{Max}-\frac{\mathrm{Min}+\mathrm{Max}}{2})$$

(2) $$+1=\frac{\mathrm{Min}+\mathrm{Max}}{2}+\frac{1}{\alpha }(\mathrm{Max}-\frac{\mathrm{Min}+\mathrm{Max}}{2})$$

Central points (0) were the medium levels of all three factors studied (0; 0; 0); they were used to check the linear relationship between the high and low levels of the variables tested (Table 4). 

The measured variable response was the R-PE extraction yield in the soluble fraction. A total of 19 experiments were carried out, corresponding to 2^3^ factorial points, 2 × 3 star points and 5 central points. Thus, the central points were performed in n = 5 (Table 1, experiments N°3, 4, 5, 11, 13) and all the other experiments were carried out in n = 1. Kinetics (0, 30, 60, 120, 180, 240, 300, 360 min) were carried out in order to follow the evolution of the R-PE extraction yields, but also to compare them with the kinetics of our previous study [35]. The order of the experiments was fully randomized. The mathematical analyses of data were carried out by the software Statgraphics Plus v.5 Experimental Design (Statgraphics Technologies Inc., The Plains, VA, USA). The accuracy of the model was further tested by conducting additional UAEH experiments.

All the optimized extractions and biochemical analyses were carried out in three independent replicates (n = 3). Means and standard deviations (SD) are given for three independent experiments. Pairwise comparisons were carried out using *t*-test (*p* < 0.05). Multiple comparison tests were carried out using the Holm–Sidak test following the ANOVA procedure (*p* < 0.05). Analyses were performed using the software SigmaStat 3.5 (Systat Software Inc., CA, San Jose, USA).

### 3.3. Conventional Extraction of R-PE

Freeze-dried *G. turuturu* was ground in liquid nitrogen to obtain a fine powder, and subsequently suspended in sodium phosphate buffer (20 mM; pH 7.1). The extraction was performed with a 1/20 ratio (*w/v*) for 20 min at 4 °C; the suspension was then centrifuged (25,000× *g*, 20 min, 4 °C) [24].

### 3.4. Analyses

#### 3.4.1. R-Phycoerythrin (R-PE)

Absorption spectra were monitored from 200 to 800 nm using a UV-VIS spectrophotometer (Shimadzu UV-1800). The R-PE concentrations were determined spectrometrically using the Beer and Eshel equation [84] (Equation (3)), where *A*_565_, *A*_592_, *A*_455_ are the absorbances at 565 nm, 592 nm, 455 nm:

[R-PE] = [(*A*_565_ − *A*_592_) − (*A*_455_ − *A*_592_) × 0.20] × 0.12 (3)

The R-PE extraction yield was expressed as mg·g^−1^ seaweed dry weight (dw).

#### 3.4.2. Determination of Seaweed Liquefaction

For the optimized conditions, the liquefaction of the material was determined over time. The proportion of soluble material was obtained, for each time, by calculating the ratio between the weight of the freeze-dried supernatant (*m*_1_) and the weight of the freeze-dried supernatant (*m*_1_) added to the weight of the freeze-dried sludge (*m*_2_) [13], expressed in percentage, according to Equation (4):

(4) $$Solubilized\;material=\frac{m_{1}}{\left(m_{1}+m_{2}\right)}\times 100$$

#### 3.4.3. Elemental Composition: Carbon and Nitrogen

The elemental C and N composition was determined on dehydrated samples: freeze-dried seaweed ground in liquid nitrogen (algal powder) and freeze-dried supernatants [13]. These dehydrated samples were weighed (1.5–5 mg) and placed in small tin capsules that were carbonized by flash combustion at 1800 °C. The C and N contents were oxidized and converted into a gaseous form, at 950 °C in a combustion column and at 750 °C in a reduction column. The gases formed were transferred by carrier gas (helium) and analysed by gas chromatography (FLASH 2000 NC Organic Elemental Analyzer—ThermoScientific, Thermo Fisher Scientific Inc., Waltham, MA, USA). The results were integrated using the Eager Xperience for Flash software Ver. 1.1 (Thermo Fisher Scientific, Inc., Waltham, MA, USA). Carbon and nitrogen extraction yields were expressed as a percentage of the initial carbon and nitrogen seaweed content (%).

#### 3.4.4. Soluble Carbohydrates

The water-soluble carbohydrates were analysed using a phenol-sulfuric acid method [85]. Glucose was used as a standard (range from to 15 to 150 mg·L^−1^) and the absorbance was measured at 490 nm (Shimadzu UV-1800, UV-VIS Spectrophotometer, Kyoto, Japan). The extraction yield of soluble carbohydrates was expressed as mg·g^−1^ seaweed dry weight (dw) [13].

#### 3.4.5. Weight-Average Molecular Weight (Mw) Determination

The weight-average molecular weight (Mw) was determined by high-performance size exclusion chromatography (HPSEC) (HPLC system Prominence, Shimadzu, Kyoto, Japan) coupled with a multiangle light scattering detector (MALS, Dawn Heleos-II, Wyatt Technology, Santa Barbara, CA, USA) and a differential refractive index (RI) detector (Optilab, Wyatt Technology, Santa Barbara, CA, USA), according to the method of Chopin et al. [86]. The samples, freeze-dried supernatants at T0 and 210 min, were dissolved in distilled water at 2 mg·mL^−1^ and filtered through a 0.45 μm cellulose acetate syringe filter before being injected.

## 4. Conclusions

This study confirmed the influence of temperature on the R-PE extracted by UAEH of *G. turuturu*. The determined optimized conditions (300 W, 20 °C, 145 L·h^−1^) for the R-PE extraction made it possible to obtain a 2.3 times higher yield (4.28 ± 0.09 mg·g^−1^ dw at 180 min) than with the conventional sodium phosphate buffer method, while avoiding expensive pre-treatments of freeze-drying and cryo-grinding. The R-PE yields reached a plateau between 90 and 210 min, and the pigment appears to be stable over time in those optimized conditions. Furthermore, after 210 min, UAEH has not only led to an increase in R-PE extraction but also in carbohydrates as well as nitrogen and carbon with extraction yields multiplied by 3.2, 1.7 and 2.1, respectively. The improved extraction of R-PE and other solubilized compounds could be attributed to the enzymatic and ultrasonic degradation of polysaccharides (Mw divided by 2.2 in 210 min), and in particular cell-wall polysaccharides, resulting in higher material transfer to the soluble phase and a potential increase in compounds preserving the R-PE. Based on these results, it would now be pertinent to thoroughly characterize and purify the R-PE, and to make an economic evaluation of this UAEH process thereafter. Finally, UAEH could thus be an efficient alternative to the conventional extraction processes currently applied on various biomasses (i.e., seaweed, microalgae, plants) that still present low extraction yields.

## Figures and Tables

**Table 1:** UAEH conditions and response for R-PE extraction yields obtained for the central composite design. Grey lines represent the central points.

Experiment N°	UAEH Conditions			R-PE Extraction Yields (mg·g^−1^ dw)							
	Power of Ultrasound (W)	Temperature (°C)	Flow Rate (L·h^−1^)	0 min	30 min	60 min	120 min	180 min	240 min	300 min	360 min
1	359	24	97	2.63	3.31	3.51	4.11	4.05	4.04	3.92	3.80
2	300	20	145	2.39	2.93	3.92	3.93	4.19	4.14	4.14	4.06
3	300	30	145	2.70	3.16	3.42	3.34	3.27	3.22	3.13	3.10
4	300	30	145	2.43	3.17	3.41	3.39	3.30	3.17	3.17	3.11
5	300	30	145	2.62	3.53	3.56	3.47	3.40	3.25	3.17	3.00
6	241	24	193	3.11	3.65	3.97	4.05	3.96	3.90	3.77	3.65
7	300	40	145	2.15	2.56	2.48	2.43	2.42	2.45	2.49	2.51
8	359	24	193	2.53	3.28	3.85	4.02	4.01	4.09	3.75	3.89
9	300	30	65	2.77	3.31	3.54	3.45	3.46	3.44	3.47	3.43
10	241	36	193	2.32	2.62	2.71	2.71	2.69	2.62	2.67	2.64
11	300	30	145	2.77	3.52	3.70	3.59	3.47	3.43	3.41	3.22
12	241	36	97	2.38	2.74	2.54	2.66	2.59	2.52	2.56	2.58
13	300	30	145	2.77	3.13	3.26	3.35	3.30	3.21	3.22	3.12
14	359	36	193	2.50	NA ^1^	2.61	2.53	2.39	2.43	2.48	2.46
15	200	30	145	2.49	3.10	2.92	3.01	2.93	2.87	2.82	2.82
16	300	30	225	2.67	3.02	3.16	3.20	3.16	3.14	3.14	2.96
17	400	30	145	2.19	3.12	3.25	3.31	3.18	3.29	3.18	3.22
18	359	36	97	2.56	2.81	2.92	2.76	2.69	2.60	2.59	2.71
19	241	24	97	2.42	3.00	3.50	3.60	3.68	3.59	3.66	3.63

^1^ Not analyzed.

**Table 2:** Equations of the models, the optimized conditions (Power (P), Temperature (T) and Flow rate (Q)) and values statistically predicted at 120, 180 and 240 min for the extraction of R-PE from *Grateloupia turuturu* using UAEH.

Time (min)	Equation of the Model	Adjusted R^2^ (%)	Optimized Conditions			Predicted Value (R-PE mg·g^−1^ dw)
			P (W)	T (°C)	Q (L·h^−1^)	
120	3.31235 − 0.535479 × T	85.46	300	20	145	4.21
180	3.33989 − 0.585277 × T − 0.0958245 × P²	92.07	300	20	145	4.32
240	3.23246 − 0.58251 × T	88.17	300	20	145	4.21

**Table 3:** Biochemical analyses conducted on soluble fractions at T0 min and T210 min of ultrasound-assisted enzymatic hydrolysis (UAEH) in optimized conditions (300 W, 20 °C, 145 L·h^−1^). These values are corrected by the amounts provided by the enzymatic cocktail. Values are means ± SD from three independent experiments (n = 3). Significant differences between T0 and T210 min are indicated by ** (*p* < 0.01), *** (*p* < 0.001).

Biochemical Analyses	Time	
	T0 min	T210 min
Nitrogen extraction yield (%)	32.88 ± 1.81	55.51 ± 1.36 ***
Carbon extraction yield (%)	22.78 ± 0.66	48.76 ± 1.53 ***
Carbohydrates (mg·g^−1^ dw)	44.31 ± 8.64	141.86 ± 20.86 **
Weight-average molecular weight (Mw) (kDa)	1080 ± 51	493 ± 24 ***

**Table 4:** Experimental design levels of independent variables used in the UAEH of *Grateloupia turuturu* (α = 1.682).

Levels of Independent Variable	Power of Ultrasound (W)	Temperature (°C)	Flow Rate (L·h^−1^)
−α	200	20	65
−1	241	24	97
0	300	30	145
+1	359	36	193
+α	400	40	225

## Data Availability

No new data were created or analyzed in this study. Data sharing is not applicable to this article.
